# Bacteriophage Bioengineering: A Transformative Approach for Targeted Drug Discovery and Beyond

**DOI:** 10.3390/pathogens12091179

**Published:** 2023-09-20

**Authors:** Longzhu Cui, Srivani Veeranarayanan, Kanate Thitiananpakorn, Dhammika Leshan Wannigama

**Affiliations:** 1 Division of Bacteriology, Department of Infection and Immunity, School of Medicine, Jichi Medical University, Tochigi 329-0498, Japan; srivani@jichi.ac.jp (S.V.); kanatethi@jichi.ac.jp (K.T.); 2 Department of Infectious Diseases and Infection Control, Yamagata Prefectural Central Hospital, Yamagata 990-2292, Japan; leshanwannigama@gmail.com

Bacteriophages, the viruses that infect and replicate within bacteria, have long been recognized as potential therapeutic agents against bacterial infections. The rise of antibiotic resistance and the limitations of traditional antibiotics have prompted researchers to explore innovative strategies in the field of drug discovery. Bacteriophage bioengineering represents a promising avenue that offers targeted and precise solutions to combat bacterial pathogens [1]. Engineered bacteriophages go beyond conventional medication, revolutionizing diagnostics by hosting illuminating genes for bacterial detection and redefining infection prevention and cancer treatment. In this editorial review, we delve into recent advances in bacteriophage bioengineering, its application in targeted drug discovery, and the potential for broader applications beyond traditional antibiotic development.

Bacteriophages possess a high degree of specificity in targeting bacterial hosts [2]. This exquisite specificity enables the precise eradication of pathogenic bacteria while sparing beneficial microbiota, thereby reducing the risk of disrupting the delicate balance in the human microbiome. The concept of bioengineering bacteriophages involves the modification of their genetic material to enhance their therapeutic potential, thereby adding desirable value to the bacteriophages. Through these modifications, one can envision well-characterized phage products. The modular bioengineering approach may include improving phage stability, increasing their host range, enhancing their bactericidal efficacy, and preventing the emergence of resistance by diversifying or modifying the phage genes, integrating added payloads, and deleting non-essential genes [3]. One of the fundamental aspects of bacteriophage bioengineering is the ability to tailor phages to target specific bacterial strains responsible for infections [4]. Researchers can develop highly targeted therapies with reduced off-target effects by selecting and engineering bacteriophages that effectively target the bacteria of interest. This approach can potentially revolutionize the treatment of bacterial infections, particularly those caused by multi-drug-resistant pathogens.

In terms of refining bacteriophages for therapeutic purposes, genetic engineering techniques have played a crucial role [5]. The manipulation of bacteriophage genomes can be achieved through various approaches, such as CRISPR-Cas gene editing, phagemid construction, and homologous recombination [6]. By incorporating genes coding for specific antimicrobial peptides or lytic enzymes, bacteriophages can be engineered to augment their bactericidal activity [7]. Additionally, the modification of phage receptors through genetic manipulation can expand their host range, enabling them to target a broader spectrum of bacterial strains [8]. Beyond their role as direct antimicrobial agents, bacteriophages harbor genes encoding enzymes with therapeutic potential [9]. Endolysins, for instance, are phage-encoded enzymes that degrade the bacterial cell wall, leading to bacterial lysis [10]. Researchers can develop innovative antimicrobial therapies that specifically target and kill bacteria by harnessing enzymes, such as lysins or biofilm-degrading enzymes. Endolysins have demonstrated efficacy against drug-resistant bacteria, highlighting their potential to combat antibiotic-resistant infections [11]. 

As bacteriophages have specific host selectivity, bioengineered bacteriophages can be used to rapidly identify pathogenic bacteria in food products via reporter or labeled nanoprobe-mediated fluorescence or luminescence-based sensing systems [12]. The purpose of engineered bacteriophages is not limited to bacterial detection and has been utilized for sensing cancer, neuropathologies, etc. [13]. Bacteriophage-based biosensors are deemed effective biosensing agents for the detection of various biomolecules with extremely high sensitivity and selectivity, encompassing diagnostic applications as well [14]. Bacteriophages can be engineered to carry reporter genes that enable the detection of specific bacterial strains or virulence factors. This novel approach could revolutionize diagnostic techniques and facilitate the rapid and accurate identification of bacterial infections, allowing for more targeted and timely treatment. Theranostics, a fusion of therapy and diagnostics, represents another fascinating avenue. By combining bioengineered bacteriophages with imaging agents, researchers can develop theranostic agents that target bacterial pathogens and provide real-time monitoring of therapeutic efficacy [15]. This integration of treatment and monitoring could potentially revolutionize infectious disease management.

The concept of utilizing bacteriophages for both vaccine development and cancer therapy demonstrates the versatility of these biological entities [16]. In the context of vaccine development, bacteriophages can be engineered to display antigens from pathogens, effectively mimicking those pathogens to stimulate an immune response. When considering cancer therapy, bacteriophage bioengineering introduces a novel avenue for precision medicine. The basic concepts behind bacteriophage bioengineering for cancer therapy is to modify bacteriophages in such a way that they can selectively internalize and destroy cancer cells while sparing normal healthy cells [17].

Although bacteriophage bioengineering holds immense promise, it is not without its challenges. One significant obstacle is the potential for the emergence of phage-resistant bacteria [18]. As bacteriophages selectively pressure bacteria, the risk of bacterial mutants evolving to evade phage predation cannot be overlooked. Thus, researchers must take a proactive approach to stay ahead of evolving bacterial resistance by continuously refining and adapting the bioengineered phages [19]. Another challenge is in terms of safety concerns. Non-replicative phages are easier to translate clinically due to their controllable nature in terms of environmental release. However, this carries a limitation of requiring repeated administration while used in therapy. However, it may still be desirable for use in specific applications as it enables precise dosing estimation in practice. Another critical aspect is the regulatory landscape surrounding bacteriophage therapies [20]. Natural phages isolated from nature cannot be patented because they are ubiquitous in availability and relatively easy to isolate with no innovative step. However, bioengineered phages with improved functionalities such as enhanced safety, antimicrobial properties, sensing ability, or any novel function can generate interest in IP protection. These features of bioengineered phages can generate interest to avail investment from pharmaceutical companies. As with any novel therapeutic approach, navigating complex regulatory routes is essential. Collaborations between researchers, pharmaceutical companies, and regulatory bodies will be pivotal in developing the translation of bacteriophage therapies from the laboratory to the clinic. Furthermore, commercializing bacteriophage-based therapies poses unique challenges compared with traditional antibiotics [21]. The targeted nature of bacteriophages may require tailored manufacturing processes for each therapeutic application. Strategies for large-scale bioengineered phage production and formulation must be optimized for efficient and cost-effective therapeutic use. In recent years, several companies have incorporated bioengineered bacteriophages into their strategic pipelines or portfolios, attracting significant venture investments for the clinical testing of these bioengineered phages.

Bacteriophage bioengineering represents an innovative approach to drug discovery, offering precise solutions for targeting harmful bacteria while preserving beneficial counterparts. Through precision engineering techniques, such as CRISPR-Cas systems, bacteriophages are equipped with specialized antimicrobial tools to enhance their efficacy and scope. This transformative field addresses challenges posed by bacterial resistance and regulatory complexities, presenting boundless potential. Furthermore, integrating treatment and real-time monitoring through theranostics reshapes infectious disease management. Bacteriophage bioengineering is at the forefront of drug discovery, influencing antibiotic resistance, diagnostics, and innovative theranostics, positioning it as a promising frontier despite obstacles. Ongoing research, requiring collaboration among researchers, pharmaceutical companies, and regulatory bodies with adaptive strategies, is crucial to address the obstacles and unlock the full potential of bioengineered bacteriophages. As highly adaptable biologics, they offer innovative disease management and precision therapeutics solutions, reshaping the healthcare landscape. The era of "Designer Phages" heralds a new chapter in addressing critical healthcare challenges [22].
